# Identification and molecular characterization of tissue-preferred rice genes and their upstream regularly sequences on a genome-wide level

**DOI:** 10.1186/s12870-014-0331-2

**Published:** 2014-11-27

**Authors:** Shu-Ye Jiang, Jeevanandam Vanitha, Yanan Bai, Srinivasan Ramachandran

**Affiliations:** Rice Functional Genomics Group, Temasek Life Sciences Laboratory, National University of Singapore, Singapore, 117604 Singapore

## Abstract

**Background:**

Gene upstream regularly sequences (URSs) can be used as one of the tools to annotate the biological functions of corresponding genes. In addition, tissue-preferred URSs are frequently used to drive the transgene expression exclusively in targeted tissues during plant transgenesis. Although many rice URSs have been molecularly characterized, it is still necessary and valuable to identify URSs that will benefit plant transformation and aid in analyzing gene function.

**Results:**

In this study, we identified and characterized root-, seed-, leaf-, and panicle-preferred genes on a genome-wide level in rice. Subsequently, their expression patterns were confirmed through quantitative real-time RT-PCR (qRT-PCR) by randomly selecting 9candidate tissue-preferred genes. In addition, 5 tissue-preferred URSs were characterized by investigating the URS::*GUS* transgenic plants. Of these URS::*GUS* analyses, the transgenic plants harboring *LOC_Os03g11350* URS::*GUS* construct showed the GUS activity only in young pollen. In contrast, when *LOC_Os10g22450* URS was used to drive the reporter *GUS* gene, the GUS activity was detected only in mature pollen. Interestingly, the *LOC_Os10g34360* URS was found to be vascular bundle preferred and its activities were restricted only to vascular bundles of leaves, roots and florets. In addition, we have also identified two URSs from genes *LOC_Os02G15090* and *LOC_Os06g31070* expressed in a seed-preferred manner showing the highest expression levels of GUS activities in mature seeds.

**Conclusion:**

By genome-wide analysis, we have identified tissue-preferred URSs, five of which were further characterized using transgenic plants harboring URS::*GUS* constructs. These data might provide some evidence for possible functions of the genes and be a valuable resource for tissue-preferred candidate URSs for plant transgenesis.

**Electronic supplementary material:**

The online version of this article (doi:10.1186/s12870-014-0331-2) contains supplementary material, which is available to authorized users.

## Background

An upstream regularly sequence (URS) is a DNA fragment upstream of a gene which acts as binding sites for transcription factors and RNA polymerases to initiate transcription. URSs play important roles in the transcriptional control of gene expression. Some of these genes are expressed throughout the life cycle of an organism, which are driven by constitutive URSs. In contrast, tissue-preferred URSs control gene expression only in a specific tissue. The activities of inducible URSs are regulated by various abiotic and biotic factors and their corresponding genes are up- or down-regulated by environmental cues or external stimuli.

It is imperative and commercially valuable to identify and characterize various types of URSs for annotating gene function by generating desired transgenic plants expressing gene of interest in a particular tissue. In eukaryotes, the URS regions are structurally more complex than those in prokaryotes. Both up- and down-stream of a transcription start site (TSS) play important roles in regulating gene expression. The TSS could be identified by aligning the full-length cDNA sequence of a gene to the corresponding genome sequence. The candidate URS sequence might be predicted by analysing around 2 Kb upstream of the start codon, which is predicted to include up- and partial down-stream region of the TSS.

In rice, the whole genome sequences of both *indica* and *japonica* subspecies had been reported [1,2] and their gene annotation systems are established [3,4]. In addition, their full-length cDNA sequence data are also available [5,6]. Thus, a bioinformatics-based approach could be employed to predict the URS sequences of all annotated genes on a genome-wide level. As a result, several URS databases have been set up and are publicly available [7-9].

Subsequent to the prediction of URS sequences, it is highly essential to further characterize these URSs’ roles in driving the transcription of the genes under their control. URS activities can be predicted by the expression profiling of their driven genes. Early studies of large-scale of expression analyses were carried out by microarrays and various chip platforms are available such as Affymetrix, Agilent, BGI/Yale, NSF20K, NSF45K and so on [10]. In addition, serial analysis of gene expression (SAGE) [11], massively parallel signature sequencing (MPSS; http://mpss.udel.edu/rice/) [12] and RNA Seq [13] have also been employed for expression analyses. Currently, large amount of data on rice gene expression have been released publicly (https://www.ebi.ac.uk/arrayexpress/; http://www.ncbi.nlm.nih.gov/geo/) [14,15]. In the meantime, various rice gene expression databases have been established. Some examples include RiceXPro (http://ricexpro.dna.affrc.go.jp/) [16], Rice Oligonucleotide Array Database (www.ricearray.org/) [13], Rice Gene Expression (http://rice.plantbiology.msu.edu/expression.shtml) [4], OryzaExpress (http://bioinf.mind.meiji.ac.jp/OryzaExpress/) [17], RicePLEX (http://www.plexdb.org/modules/PD_browse/experiment_browser.php?experiment=OS5) [18] and rice expression database (http://cdna02.dna.affrc.go.jp/RED/) [19]. In addition, the genome-wide expression analysis was also carried out to dissect the rice gene expression profile. Several reports have focused on the expression analysis of genes in multiple tissues and developmental stages. Jain et al. [20] carried out the rice Affymetrix microarray analysis using 15 different tissue samples at various developmental stages. Wang et al. [21] carried out a dynamic gene expression profile covering the entire life cycle of rice. They also employed the Affymetrix Genechips to investigate the rice gene expression using 39 tissues at various developmental stages. Sato et al. [22] carried out a transcriptome analysis using 48 tissue samples and showed critical developmental and physiological transitions throughout life cycle of rice growing under natural field conditions. Besides microarray analysis, Nobuta et al. [12] used the MPSS to analyze rice gene expression by sequencing mRNA transcripts from 22 libraries and revealed new expression evidence of some genes in which no expression signal was previously detected. In addition, Davidson et al. [23] carried out transcriptome analysis using 12 rice tissues from various developmental stages by the RNA_Seq technology, providing additional resources of rice gene expression data. Although large amount of expression data are available, relatively limited reports focused on the investigation of tissue-preferred gene expression patterns.

In rice, a considerable number of URSs have been isolated and characterized. Some of them have been used for driving the constitutive expression of a foreign gene in transgenic plants. Examples include the URSs for the genes *OsAct1* [24], *OsCc1* [25] and *OsRUBQ1* [26]. Others are root-preferred [27-29], leaf-preferred [30-32], panicle-preferred [33-35] or seed-preferred [36-38]. Although many rice URSs have been molecularly characterized, it is still necessary and useful to identify various types of URSs on a genome-wide level to benefit researchers in plant transformation and gene function annotation. In this study, we had identified various types of tissue-preferred genes based on their expression patterns on a genome-wide level. Subsequently, a few URSs were selected and cloned into upstream of the *uidA* gene, which encodes β-glucuronidase (GUS) to investigate their transcription activities through GUS expression. Our results provide 5 tissue-preferred candidate genes for sourcing their URSs, which may be useful for gene function annotation and plant transformation for genetic improvement.

## Results

### Genome-wide survey of tissue-preferred genes in the rice genome

**Figure 1:**
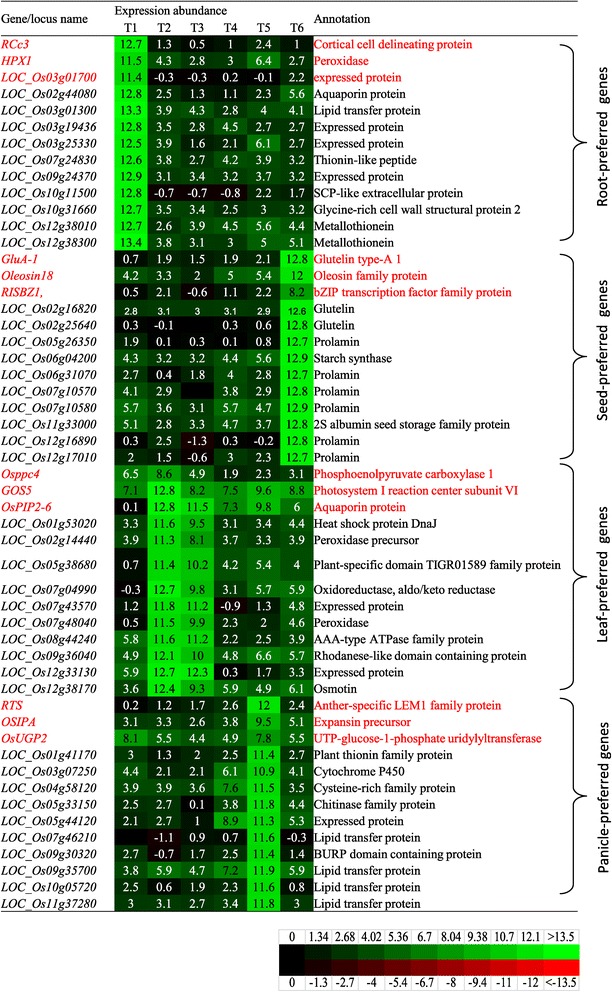
**Tissue-preferred genes and their expression profiling among various developmental stages of tissues.** A total of 13 genes were listed in each group of tissue-preferred genes. The first three genes in each group were formatted with red fonts, which were previously characterized and, therefore were used as reference genes. The remaining 10 genes were formatted with black fonts, which were identified in this study. The log_2_-transformed expression value from normalized expression data were used for heat map analyses. Red, black, and green colors indicated that transformed expression values were <0, = 0, and >0, respectively, in the matrix. T1, roots; T2, mature leaves; T3, young leaves; T4, young inflorescence (up to 3 cm); T5, inflorescence (3–30 cm); T6, seeds.

To investigate tissue-preferred genes in the rice genome, related microarray, MPSS and RNA_Seq expression data were downloaded from the GEO dataset as described in the Methods section. Initially, we employed the dataset with accession number GSE6893 [20] to identify the following 4 types of genes: (1) root-preferred, (2) seed-preferred; (3) leaf-preferred; and (4) panicle-preferred genes. The expression patterns of these candidate tissue-preferred genes were verified by the remaining three expression datasets as indicated in the Methods. Genes with inconsistent expression patterns among different datasets were excluded from further analysis. Using this criteria, we have identified 94 root-preferred (Additional file 1), 83 seed-preferred genes (Additional file 2), 63 leaf-preferred genes (Additional file 3), and 30 panicle-preferred genes (Additional file 4). For each type of tissue-preferred genes, 10 genes were selected for further analysis (Figure 1). Among the 10 selected root-preferred genes, most of them also showed higher or similar expression abundance in roots when compared with three previously identified genes *RCc3* [27], *HPX1* [29] and *LOC_Os03g01700* [28] while no or very low expression was detected in the remaining tissues (these with red fonts are known reference genes and those genes with black fonts are new from this study in Figure 1). For the three previously identified leaf-preferred genes *Osppc4* [32], *GOS5* [30], and *OsPIP2-6* [31], they were expressed in leaf with higher level but they also showed significant expression in other tissues. In contrast, 10 selected leaf-preferred genes were mainly detected in mature and young leaves and no or very low signal could be detected in the remaining tissues. As expected, three previously identified panicle-preferred genes *RTS* [33], *OSIPA* [35], and *OsUGP2* [34], *RTS* showed very high expression in panicles (Figure 1). In this analysis, we identified only 30 panicle-preferred genes (Additional file 4). Out of these, ten genes were listed and all of them showed similar expression level in panicles compared to that of a previously identified panicle-preferred *RTS* but higher than the expression level of other two previously identified panicle-preferred *OSIPA* and *OsUGP2* (Figure 1).

Tissue-preferred genes are mainly expressed in a particular tissue or cell type. Their functions may be restricted to the tissue or cell type. To evaluate whether these genes are biased toward particular functions, we investigated Gene Ontology (GO) terms [39] and identified overrepresented GO terms (Additional file 5) in all four types of expressed genes. A total of three categories of GO terms have been assigned to these genes including molecular function (F), biological process (B), and cellular component (C) [39]. Overrepresented root-preferred genes were found to play roles in response to stress and transport (for Biological Processes); they are mainly localized on cell wall, membrane or cytoplasm with hydrolase, transporter and catalytic activities as well as for lipid and RNA binding (yellow columns in Additional file 5A). In contrast, for seed-preferred genes, their biological functions in “multicellular organismal development” and “developmental process” were overrepresented (blue column in Additional file 5B). On the other hand, overrepresented leaf-preferred genes are mainly localized in plastid, membrane, thylakoid, cytoplasm, organelle or intracellular and their overrepresented molecular function is catalytic activity (green columns in Additional file 5C). For panicle-preferred genes, their overrepresented GO terms included “transport”, “establishment of localization”, “secondary metabolic process”, “cellular amino acid”, “derivative metabolic process”, “small molecule metabolic process and “lipid binding” for molecular function (brown columns in Additional file 5D).

### Expression analysis 9 candidate endogenous genes in 11 rice tissues

**Figure 2:**
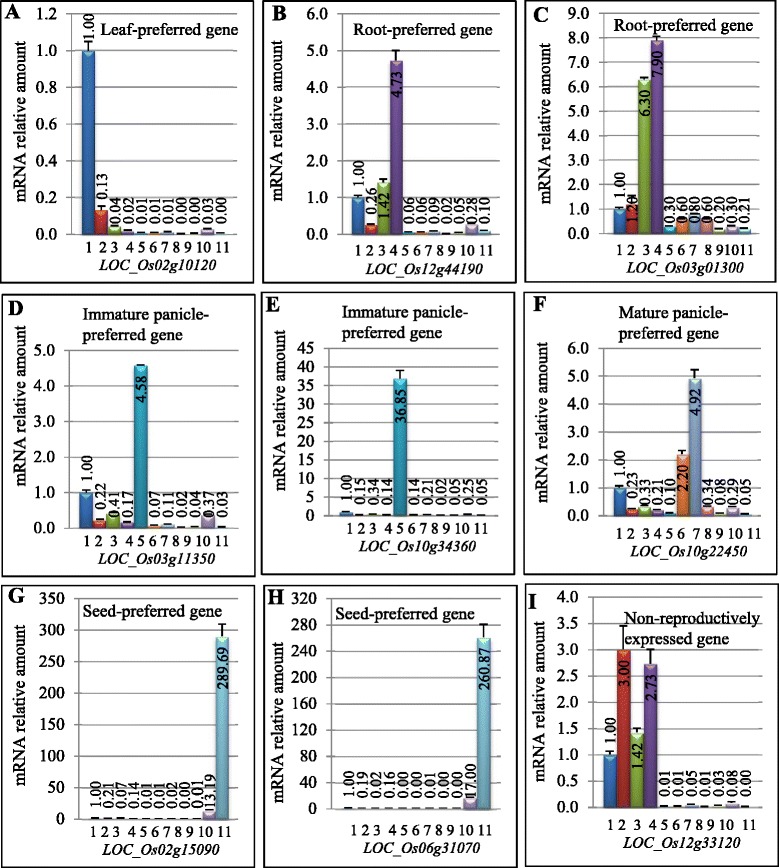
**Expression patterns of some tissue-preferred genes in various tissues shown by qRT-PCR analysis.** The mRNA relative amount was calculated as described in the section “Methods”. **(A)** to **(I)** showed the expression patterns of 9 tissue-preferred genes. The total RNA samples were prepared from a total of 11 tissues at different developmental stages, which were used as templates for qRT-PCR. These tissues were shown as below: 1, two-week old leaves; 2, two-month old leaves; 3, two-week old roots; 4, two-month old roots; 5, 0-5 cm long panicles; 6, 5-10 cm long panicles; 7, more than 10 cm long panicles; 8, opening panicles; 9, flowering panicles; 10, milky seeds; and 11, mature seeds (21 days after pollination).

By genome-wide survey of tissue-preferred genes using microarray, MPSS or RNA_Seq analysis, we have identified considerable numbers of genes with tissue-preferred expression. To verify the expression of these genes, 9 genes were randomly selected for quantitative real-time RT-PCR (qRT-PCR) analysis to investigate their expression profile among 11 different tissues as shown in Figure 2. The qRT-PCR expression data confirmed the tissue-preferred expression patterns when compared with the available expression data from microarray, MPSS or RNA_Seq. For example, the gene *LOC_Os02g10120*, encoding a lipoxygenase, was found to be leaf-preferred and was mainly expressed in two-week old leaves (Figure 2A). The gene *LOC_Os12g44190*, encoding ATPase 3, was root-preferred with the highest expression in two-month old roots (Figure 2B). Another root-preferred gene *LOC_Os03g01300* encodes protease inhibitor and was mainly expressed in young and mature roots (Figure 2C). For panicle-preferred genes, we selected 3 genes for expression validation. Both *LOC_Os03g11350* and *LOC_Os10g34360* encode UDP-glucosyltransferase and stilbene synthase, respectively. They showed immature panicle-preferred expression with the highest expression level at the 5–10 cm length stage of panicles (Figure 2D and E). The remaining one gene *LOC_Os10g22450* encodes inositol-3-phosphate synthase, which was mainly expressed in more than 10 cm panicles that were wrapped inside leaf sheath (Figure 2F). For two seed-preferred genes, both of them were mainly expressed in mature seeds (21 days after pollination, Figure 2G and H). The gene *LOC_Os02g15090* encodes glutelin and *LOC_Os06g31070* encodes a prolamin precursor. The gene with locus name *LOC_Os12g33120* encodes an expressed protein with unknown function. Its expression was detected only in leaves and roots but not in reproductive tissues (Figure 2I).

### The gene *LOC_Os03g11350* showed expression mainly in young pollen

**Figure 3:**
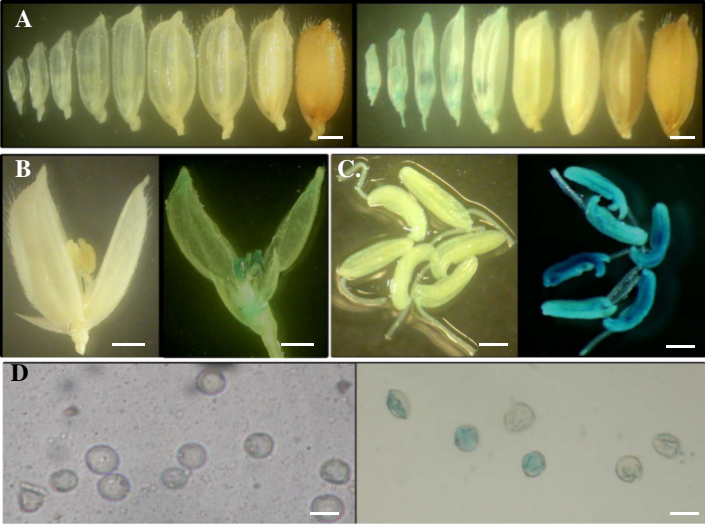
**GUS activities in the URS::** ***GUS*** **transgenic plants for the gene with locus name** ***LOC_Os03g11350*** **. (A)** Different stages of rice florets/seeds. **(B)** Enlarged rice florets. **(C)** Enlarged rice young anthers. **(D)** Pollen at the uni-nucleate stage. In **(A)** to **(D)**, left and right images were from WT and the transgenic plants, respectively. Bars: 1 mm in **(A)** to **(C)** and 50 μm in **(D)**.

Our data from qRT-PCR analysis showed that the gene *LOC_Os03g11350* was mainly expressed at the early stage of panicle development (Figure 2D). To further investigate the expression patterns at the cellular level, we generated the URS::*GUS* (encoding β-glucuronidase) transgenic plants. For each gene, around 2 Kb URS region upstream of start codon of the gene was used for URS motif searches and primer selection. For the gene *LOC_Os03g11350*, the 1,805 bp URS fragment was amplified from the rice genome using the primers listed in the Additional file 6. The fragment was subsequently cloned upstream of the reporter *GUS* gene. Following the cloning, this construct was transformed into the rice genome by *Agrobacterium*-mediated transformation. The investigation on the URS::*GUS* plants showed that no GUS activity was observed in leaves or roots or any other non-reproductive tissues. The GUS activity was detected only at the early stage of panicle development (Figure 3A). Further investigation showed that the GUS activity was limited only to the anthers but not in the floret husks (Figure 3B and C). The GUS-stained anthers were then squeezed with a forceps and pollen was subjected to further observation under microscope. The result showed that the activity of the URS was restricted to young pollen at the uninucleate stage (Figure 3D, data not shown in the other stages). The qRT-PCR was carried out to analyze expression abundance of the *GUS* reporter gene and the result confirmed that the gene *LOC_Os03g11350* was mainly expressed in 0–5 cm long immature panicles (Additional file 7A).

### The gene *LOC_Os10g34360* showed vascular bundle preferred expression by its URS::GUS activity analysis

**Figure 4:**
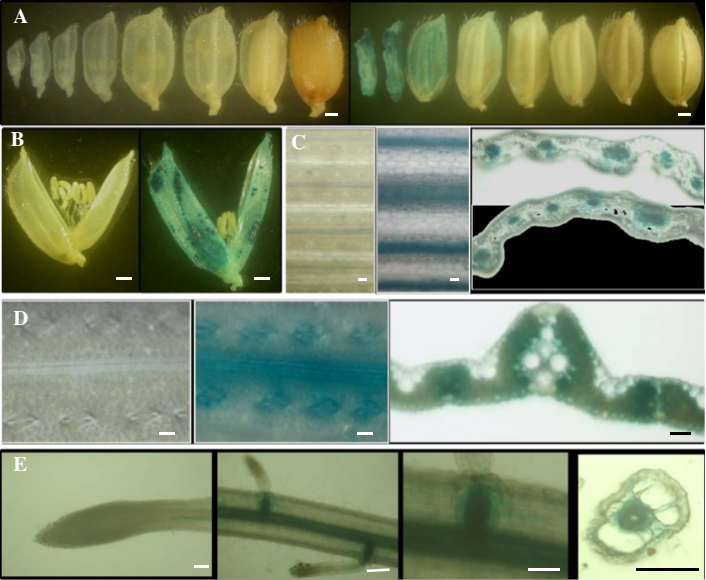
**GUS expression patterns of the URS::** ***GUS*** **transgenic plants for the gene with locus name** ***LOC_Os10g34360*** **. (A)** Different stages of rice florets/seeds. **(B)** Enlarged rice florets. **(C)** leaves. Left, WT; middle, the transgenic leaf; right, cross section of the transgenic leaf. **(D)** Leaf veins. Left, WT; middle, the transgenic leaf vein; right, cross section of the transgenic leaf vein; **(E)** Roots. Left, the whole root; middle, vertical sections of roots; right, cross section of the root. Bars: 0.5 mm.

Similar to the gene *LOC_Os03g11350*, the endogenous gene *LOC_Os10g34360* was also mainly detected at the early stage of panicle development as shown by qRT-PCR (Figure 2D and E). The URS::GUS activity was also observed at the early stage of florets (Figure 4A). However, while no GUS staining was observed in anthers and the staining was restricted only to floret husks (Figure 4B). Although the gene *LOC_Os10g34360* was mainly expressed in panicles (Figure 2E), the URS also showed activities in both leaves and roots (Figure 4C-E). Interestingly, either in leaves or in roots, the GUS activities were detectable only in vascular bundles, similar to the expression patterns in floret husks. Thus, the gene showed vascular bundle preferred expression. The GUS activities in both leaves and roots were also in according with the qRT-PCR analysis as shown in the Additional file 7B.

### The gene *LOC_Os10g22450* was mainly expressed in mature pollen

**Figure 5:**
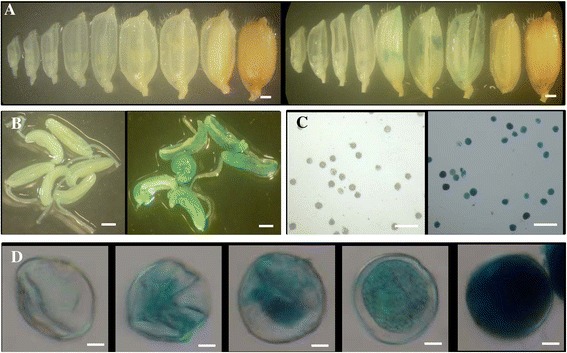
**Pollen-preferred GUS activities in the URS::** ***GUS*** **transgenic plants for the gene with locus name** ***LOC_Os10g22450*** **. (A)** Different stages of rice florets/seeds. **(B)** Enlarged rice anthers. **(C)** Pollen. **(D)** Different developmental stages of pollen. From **(A)** to **(C)**, left and right images were from WT and the transgenic plants, respectively. Bars: 0.5 mm in **(A)** and **(B)**; 200 μm in **(C)**; 10 μm in **(D)**.

Based on the qRT-PCR data, the gene *LOC_Os10g22450* showed the highest expression level at the panicle with more than 10 cm long and the gene also showed the high expression level at the 5–10 cm long panicles (Figure 2F). A similar expression pattern was observed in the transgenic plants harboring its URS::*GUS* construct as the GUS activity was only detected in the florets of panicles with more than 10 cm long (Figure 5A). Further observation showed that GUS staining was restricted to anthers and no GUS activity was observed in lemma and palea of rice florets (Figure 5B). Under microscope, the GUS activity was observed only in pollen but not in anther walls (Figure 5C). Further examination showed that the faint GUS activity could be detected from the uni-nucleate stage of pollen and the strongest activity was observed at the mature stage of pollen (Figure 5D). However, no GUS activity was detected in pollen tubes. The qRT-PCR analysis of the *GUS* reporter gene further confirmed that the gene *LOC_Os10g22450* was mainly expressed in the mature pollen (Additional file 7C).

### The seed-preferred URS from the gene *LOC_Os02g15090*

**Figure 6:**
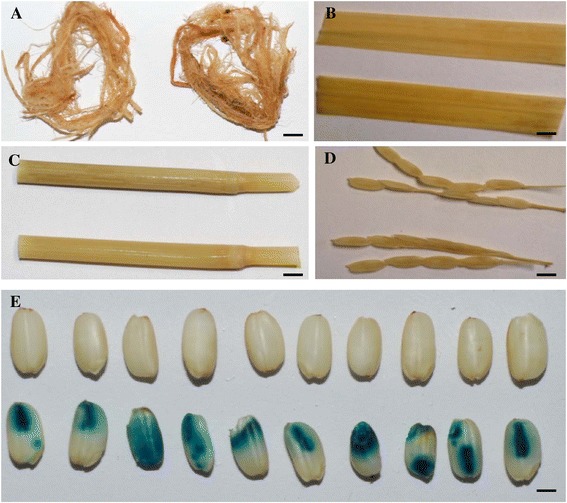
**Seed-preferred GUS activities in the transgenic plants carrying the** ***LOC_Os02g15090*** **URS::** ***GUS*** **construct. (A)** Roots. Left, WT root; Right, the transgenic root. **(B)** Leaves. **(C)** Stems. **(D)** Panicles. **(E)** Mature seeds. From **(B)** to **(E)**, the top image were from WT and the bottom images were from the transgenic plants. Bars: 5 mm in **(A)** to **(D)** and 1 mm in **(E)**.

The qRT-PCR analysis showed that the gene *LOC_Os02g15090* showed seed-preferred expression (Figure 2G). A 1,839 bp of URS sequence of this gene was isolated from the rice genome and this region was found to contain two seed-preferred motifs including *AACA*_motif and *Skn-1*_motif [40,41]. The former motif was shown to play a role in suppressing the expression of this gene in other tissues other than endosperm. The latter is a *cis*-regulatory element along with cooperative interaction with other motifs such as *AACA*, *GCN4* and *ACGT*, required for high level of endosperm expression of this gene. The transgenic plants harboring the URS::*GUS* T-DNA showed no GUS activity in leaves, stems, roots and panicles (Figure 6A-D). In contrast, the GUS activity was detected only in seeds (Figure 6E). Upon further examination, the GUS expression as indicated by the staining was observed in endosperm as well as embryos (Figure 6E). Subsequently, we quantified the expression abundance of the reporter *GUS* gene in various tissues by qRT-PCR analysis. The results showed that the *GUS* gene exhibited the highest transcript abundance in mature seeds (Additional file 7D).

### The *LOC_Os06g31070* gene also shows seed-preferred URS activity

**Figure 7:**
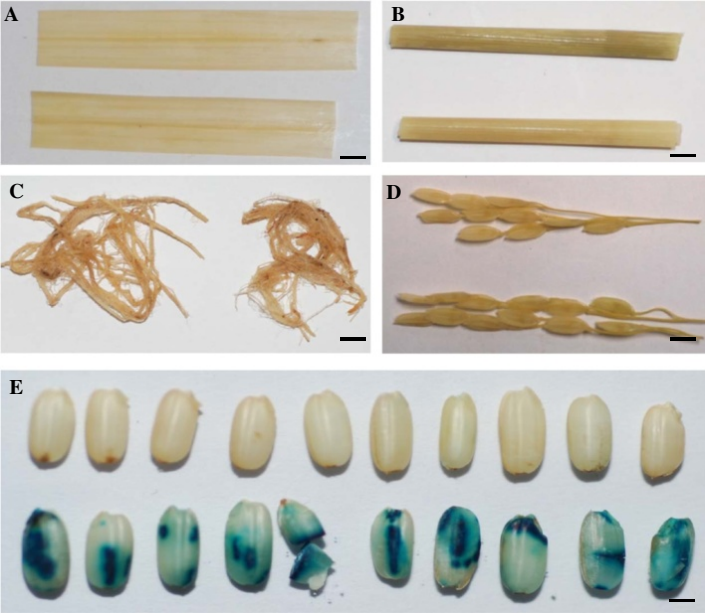
**Seed-preferred URS activities shown by the transgenic plants carrying the** ***LOC_Os06g31070*** **URS::** ***GUS*** **construct. (A)** Leaves from WT (top) and the transgenic plants (bottom). **(B)** Stems from WT (top) and the transgenic plants (bottom). **(C)** Roots from WT (Left) and the transgenic plants (right). **(D)** Panicles from WT (top) and the transgenic plants (bottom). **(E)** Mature seeds from WT (top) and the transgenic plants (bottom). Bars: 5 mm in **(A)** to **(D)** and 1 mm in **(E)**.

Besides the seed-preferred URS from the *LOC_Os02g15090* gene, we have also investigated another URS, which drives the expression of the *LOC_Os06g31070* gene. The qRT-PCR analysis showed that this gene was also mainly expressed in seeds (Figure 2H). A 1,678 bp long URS fragment of this gene was amplified by PCR and was subjected to sequencing confirmation. The sequencing analysis showed that its URS possessed only one seed-related motif, *Skn-1*_motif. Interestingly, the transgenic rice plants harboring the URS::*GUS* construct showed similar expression pattern to its endogenous gene by qRT-PCR) with no GUS activity in roots, leaves, stems and panicles (Figure 2H and Figure 7A-D). In contrast, GUS activity was observed in seeds including endosperms and embryos (Figure 7E). As expected, the highest expression of the reporter *GUS* gene in mature seeds was further confirmed by qRT-PCR analysis (Additional file 7E).

## Discussion

### Candidate tissue-preferred genes and their URSs for the area of transgenesis

Tissue-preferred genes provide candidate URSs for transgenic plant development. We have identified a considerable number of tissue-preferred genes which are either vegetative (leaf/root) or reproductive (panicle/seed) tissue preferred. Not all tissue-preferred genes were listed in this study. Some of tissue-preferred genes were not included due to their relatively low expression level in that specific tissue. The tissue-preferred URSs that are highly expressed will be used for functional genomics studies and genetic modification of crops by transgenic techniques. The number of characterized tissue-preferred URSs from monocot plants is less than those from dicot plants [42]. In addition, many of these tissue-preferred URSs have been patented, limiting their use in biotechnology crop modification [43,44]. Our data provides additional resources to further characterize novel URSs for tissue-preferred expression of targeted genes which will benefit crop breeding approaches that use transgenic techniques.

### Messenger RNA-level expression and tissue/cell-level reporter gene analysis in transgenic rice plants

By the genome-wide survey of gene expression level among multiple tissues, we have identified a considerable number of genes with leaf-preferred, root-preferred, panicle-preferred and seed-preferred expression patterns. However, the activities of their URSs are required to be verified by the reporter gene analysis in transgenic rice plants. Our data showed that even for these genes with the same tissue specificity by mRNA level expression analysis, they may exhibit difference in their expression patterns in tissue/cell level. For example, both genes *LOC_Os03g11350* and *LOC_Os10g22450* are panicle-preferred; the URS::GUS analysis showed that the former was young pollen preferred (Figure 3) and the latter was mature pollen preferred (Figure 5). Thus, the activity of an URS must be confirmed by the corresponding URS driven reporter gene in their transgenic plants. On the other hand, the expression profile of an internal gene revealed from mRNA expression data may be different from that of the reporter gene. One of the examples is the gene *LOC_Os10g34360*. The gene exhibited panicle-preferred expression pattern and its activity was mainly detected in the inflorescence with 3–30 cm long (Additional file 4). However, in the transgenic plants harboring its URS::*GUS* cassette, the GUS activities could be detected not only in the floret husks but also in the vascular bundles of leaves and roots (Figure 4). The data suggested that an URS from a panicle-preferred gene might also drive the expression of the reporter gene in non-panicle tissues. However, further investigation should be carried out to figure out the inconsistency of expression patterns between endogenous mRNA and the reporter *GUS* gene.

### Tissue-preferred genes and their functions

In rice, at least 31,382 genes showed expression evidence by microarray, cDNA/EST, and MPSS [45]. More genes were detected with expression signal by custom microarray analysis [46]. In this study, we have identified various types of tissue-preferred genes. We have detected multiple overrepresented GO terms in each type of tissue-preferred genes by Gene Set Enrichment Analysis (GSEA, see Methods). The results suggested that these genes might play certain roles which should be required for tissue-preferred functions. Thus, tissue-preferred gene expression patterns were often used as a reference to identify functionally relevant genes [47]. Protein domain analysis showed that many seed-preferred genes encode glutelins, cupin domain containing proteins, late embryogenesis abundant proteins, prolamins, and seed allergenic proteins and many of these proteins are mainly accumulated during seed development. Thus, they showed seed-preferred expression. Similar situations were also observed in leaf-, root-, and panicle-preferred genes. For example, the rice plastid sigma factor *OsSIG1* (*LOC_Os08g06630*) is a leaf-preferred gene (Additional file 3) and its expression in leaves plays a role in the maintenance of photosynthetic activity [48]. The gene *RST2* (*LOC_Os01g70440*) was required for rice male fertility [33] and therefore was only expressed in panicles (Additional file 4). Thus, tissue-preferred expression of genes would avoid unnecessary bioenergy waste which was due to the gene transcription in other tissues.

### Motifs from tissue-preferred genes and synthetic URSs

Sometimes, endogenous plant URSs are not strong enough for plant transformation to obtain desirable phenotypes. By contrast, synthetic URSs can be designed to be stronger. They can also be used as regulatory devices for controlling constitutive, inducible, tissue-preferred gene expression [49]. Currently, most of synthetic URSs were generated by inserting functional motifs into natural URSs [49]. For example, the higher level activities of URSs Pcec [50] and Mac [51] were constructed by introducing enhancer motifs into the upstream of native constitutive URSs. Although considerable synthetic URSs have been generated [49], most of them were constitutive or inducible URSs. Relatively, much less was reported on synthetic tissue-preferred URSs. By investigating the overrepresented URS motifs in leaf-, root-, panicle- and seed-preferred genes, we have identified at least one tissue-preferred URS motifs. These motifs include *GCnGCnGC* for leaf-specificity, *GCTAGCTA* for root-specificity, *AnwATATA* for panicle-specificity and *yATATnTT* for seed-specificity (Additional file 8A-D). They were overrepresented in corresponding tissue-preferred URSs. We have also further analysed the known tissue-preferred motifs for two panicle-preferred and two seed-preferred URSs (Additional file 8E-G). Thus, these motifs provide candidates for designing new tissue-preferred URSs. On the other hand, the identification of these tissue-preferred URSs and their motifs will benefit not only the designing of synthetic URSs but also the computer prediction of expression patterns of genes in other closely related species. These, in return, may provide a reference for function annotation of these genes in the species.

## Conclusions

In this study, we have genome-widely identified root-, leaf-, panicle- and seed-preferred genes in the rice genome by comparing the expression abundance among different rice tissues. Some of these tissue-preferred genes were verified through qRT-PCR expression analysis. Based on these analyses, we have identified 94 root-preferred, 83 seed-preferred, 63 leaf-preferred and 30 panicle-preferred genes. In addition to these, a total of 5 URSs were isolated and their activities were further investigated by analyzing transgenic rice plants harboring the URS::*GUS* cassettes. The transgenic analysis revealed one young pollen preferred, one mature pollen preferred, one vascular bundle preferred and two seed-preferred URSs. Thus, our data might provide some evidence for gene function annotation and candidate URSs for plant transgenesis.

## Methods

### Plant materials and growth conditions

Nipponbare (japonica) rice plants (*Oryza sativa* L.) were used for all experiments. More information about the cultivar “Nipponbare” is available at the National Plant Germplasm Systems of the USDA Agricultural Research Service (http://www.ars-grin.gov/npgs/) with accession number PI 514663. Seeds were germinated in water at 37°C for 3 days and the germinated seeds were planted in greenhouse and were grown under natural light and temperature conditions in Singapore.

### Isolation of tissue-preferred URSs and construction of the URS::*GUS* cassettes

Around 2 Kb of regularly sequences upstream of the start codon of tissue-preferred genes including 5′-untranslated region (UTR) were retrieved from the release 7 of MSU (Michigan State University) Rice Genome Annotation Project Database (http://rice.plantbiology.msu.edu/index.shtml) [4]. The putative URSs were then submitted to the promoter databases PlantCare (http://bioinformatics.psb.ugent.be/webtools/plantcare/html/) [52] and PLACE (http://www.dna.affrc.go.jp/PLACE/) [53] for motif searches. The searches formed the basis for primer design to cover possible tissue-preferred motifs. Finally, primers were selected by the PrimerSelect program from DNASTAR Lasergene 10 core suit (http://www.dnastar.com/) [54] and were used to amplify the URS fragments from genomic DNA. All primer sequences were listed in the Additional file 6. PCR amplifications were carried out in 25 μl reaction mixtures with 50 ng of genomic DNA, 200 μM of each of dNTPs, 0.5 μM each of primers, 2.5 mM MgCl_2_, 1 unit of DNA polymerase, and buffer provided by the polymerase supplier Qiagen. The reactions were performed in PTC100 (MJ Research, Inc.) thermocycler starting with 94 for 5 min followed by 30 cycles at 94°C for 40 s, 55°C-68°C for 1 min (depending on the Tm value of primers) and 72°C for 2 min. The reactions were terminated with a 10 min extension step at 72°C. The amplified fragments were purified from agarose gel for sequencing. After verification, the fragments were then cloned into the pGEM®-T Easy Vector (www.promega.com) for subcloning. The backbone vector used in this study was pCambia 1301 (http://www.cambia.org/). In this vector, the *GUS* gene was driven by the 35S promoter. We developed the tissue-preferred URS::*GUS* constructs by replacing the 35S promoter with the tissue-preferred URSs. In the backbone vector, *NOS* terminator was used for the *GUS* reporter gene. The *HPT* gene encoding hygromycin phosphotransferase was used for selection, which was driven by CAMV35S promoter and was terminated by CaMV 3′UTR (polyA signal, around 200 bp).

### Generation of transgenic rice plants harboring the URS::*GUS* cassettes

Constructs were first introduced into *Agrobacterium* strain *AGL1* by electroporation using GIBCO-BRL Cell-Porator. After confirmation by mini-preparing plasmid DNA samples from the *Agrobacteria* followed by restriction enzyme digestion, the transformed *Agrobacteria* were used for rice transformation according to the protocol reported by Hiei et al. [55].

### URS analysis of tissue-preferred genes by GUS staining

GUS histochemical staining solution was prepared with 0.02 M 5-bromo-4-chloro-3-indolyl-bb-D-glucuronide, 0.1 M NaH_2_O_4_, 0.25 M ethylenediaminetetraacetic acid (EDTA), 5 mM potassium ferricyanide, 5 mM potassium ferrocyanide, and 1.0% (v/v) Triton X-100. The solution was adjusted to pH 7.0. Various tissues at different developmental stages were collected and were placed into the GUS staining solution. After incubation at 37°C for overnight in the staining solution, tissues were decolorized by 70% alcohol. Nikon microscope was used for the observation of GUS activity.

### Quantitative real-time RT-PCR (qRT-PCR)

For qRT-PCR analysis, various tissues from different developmental stages were collected, including two-week old leaves and roots, two-month old leaves and roots, 0-5 cm long panicles, 5-10 cm long panicle, more than 10 cm long panicles, opening panicles, flowering panicles, milky seeds and mature seeds. Samples were first frozen in liquid nitrogen and were then used for RNA extraction using RNeasy Plant mini kit (Qiagen). All primers used for qRT-PCR were designed by Applied Biosystems (AB) Primer Express software. Designed primer sets were then submitted to the NCBI database for BLAST searches to eliminate non-preferred primers. Gene-specific primer sequences were listed in the Additional file 6.

The qRT-PCR analyses were performed using AB 7900HT PCR system 384 well formats. Each reaction was performed using the AB power SYBR Green PCR Master mix kit (P/N 4367659) according to the manufacturer’s protocol. The reactions were denaturized at 95°C for 10 min, followed by 40 cycles of denaturation at 95°C for 15 s and annealing/extension at 60°C for 1 min. Two biological replicates and technical triplicates for each replicate were carried out for all analyzed genes. The rice *eEF-1a* gene was used as an internal control to normalize the expression data and its primer sequences were listed in the Additional file 6. The threshold cycle (C_T_) value was automatically calculated by the ABI 7900 system software. The ∆C_T_ and ∆∆C_T_ value were calculated according to Jiang et al. [56]. The mRNA relative amount (2^-∆∆CT^) was used for chart preparation.

### Databases used in this study and identification of tissue-preferred genes

Four datasets were used to identify tissue-preferred genes. One of them was the MPSS database (http://mpss.udel.edu/rice/) [12]. The data normalization and signatures matching in the rice genome were according to the method by Nobuta et al. [12]. Other two datasets were from the NCBI GEO database with accession numbers GSE6893and GSE19024 [21] for Affymetrix microarray analysis. The raw data normalization was carried out according to the description by Wang et al. [21]. The remaining one dataset was from RNA_Seq with accession number GSE16631 [57]. The normalized expression data were downloaded from the MSU Rice Genome Annotation database (release 7; http://rice.plantbiology.msu.edu/index.shtml). All the gene annotation and URSs were also downloaded from the MSU Rice Genome Annotation database. Both the PLACE and PlantCare databases were used to analyze known URS motifs.

We first used the expression dataset GSE6893 to identify all tissue-preferred genes. For the identification of root-, seed-, leaf- or panicle-preferred genes, the expression abundance in the preferred tissue should be at least 10 times higher than the expression abundance in any of the remaining tissues and their expression level showed the significant difference by Student’s *t*-test at P <0.05. After the identification of all putative tissue-preferred genes from the dataset GSE6893, their expression patterns were further confirmed by using the remaining 3 datasets as mentioned in this paragraph. All putative tissue-preferred genes with inconsistent expression patterns among the four datasets were excluded for further analysis.

### GO assignment, annotation and gene set enrichment analysis

Plant GOSlim ontologies have been assigned to the annotated rice proteins in the release 7 of the MSU dataset [4]. A total of 34,314 models in the release 7 of the database have been assigned Gene Ontologies (http://rice.plantbiology.msu.edu/index.shtml). We obtained GO assignments for rice genes in the database. Gene Set Enrichment Analysis (GSEA) [58] was used to determine if a GO category was over-represented in tissue-preferred genes. GSEA was carried out by statistically comparing the partition of the GO category in a group of targeted genes with that in all annotated rice genes with p < 0.05 and false discovery rate (FDR) <0.25.

### Detection and prediction of URS motifs and their overrepresentation analysis

The whole rice genome sequence was downloaded from the release 7 of MSU rice genome annotation database [4] for sequence extraction of URSs. A total of 2-kb upstream of start codon of each gene was retrieved from the genome for motif detection and prediction. Known URS motifs were detected by the PLACE and PlantCare programs. The BioProspector program [59] was used to detect overrepresented motifs. For running the BioProspector program, the motif width was set to 8 bp and all rice URSs 2-kb upstream of start codon of annotated genes were used as the background sequences. All other parameters were from default sets for the program. We selected only one overrepresented motif for each set of URSs, which had the highest MotifScore. We used the enoLOGOS program [60] to generate URS logos of detected URS motifs.

### Availability of supporting data

The data sets supporting the results of this article are included within the article and its additional data files.

## Additional files

Additional file 1:
**Root-preferred genes in rice.**
Additional file 2:
**Seed-preferred genes in rice.**
Additional file 3:
**Leaf-preferred genes in rice.**
Additional file 4:
**Panicle-preferred genes in rice.**
Additional file 5:
**GO term analysis of gene functions and GSEA.**
Additional file 6:
**Primer sequences used for URS amplification and qRT-PCR.**
Additional file 7:
***GUS*** **gene expression patterns of transgenic lines carrying a URS::** ***GUS*** **cassette shown by qRT-PCR analysis.**
Additional file 8:
**Overrepresented URS motifs.**

## Abbreviations

CT: Threshold cycle
EDTA: Ethylenediaminetetraacetic acid
EST: Expressed sequence tag
FDR: False discovery rate
GSEA: Gene set enrichment analysis
GUS: β-glucuronidase
MPSS: Massively parallel signature sequencing
qRT-PCR: Quantitative real time reverse transcription PCR
SAGE: Serial analysis of gene expression
