# Supplementary material for: Identification and molecular characterization of tissue-preferred rice genes and their upstream regularly sequences on a genome-wide level
Source: BMC Plant Biol. 2014 Nov 27;14:331. doi: 10.1186/s12870-014-0331-2 (PMC4248441; doi:10.1186/s12870-014-0331-2)
Supplement: Additional file 5: — GO term analysis of gene functions and GSEA. [file 12870_2014_331_MOESM5_ESM.pdf]

Additional data file 5

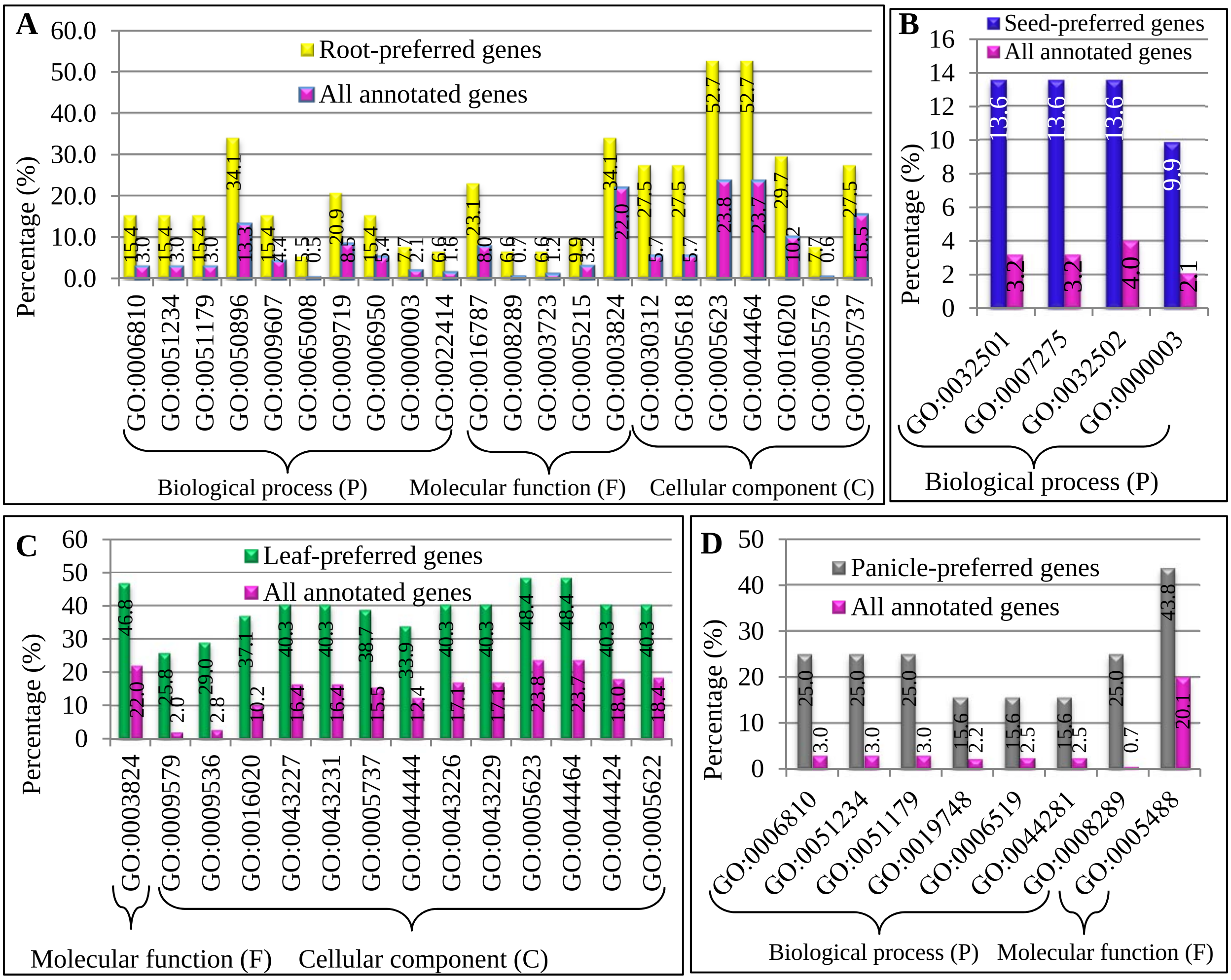

Additional data file 5. GO term analysis of gene functions and GSEA.

(A) to **(D)** GO term analysis of proteins deduced from root-, seed-, leaf- and panicle-preferred genes, respectively. In **(A)** to **(D)**, pink columns indicate the percentages of this GO term in total annotated rice proteins in the release 7 of MSU rice genome annotation database (http://rice.plantbiology.msu.edu/). Yellow, blue, green and grey columns indicate the percentages of this GO term in proteins deduced from root-, seed-, leaf- and panicle-preferred genes, respectively. Only GO terms with statistically significant differences at P<0.05 were listed in **(A)** to **(D)**. GO term annotation in **(A)** to **(D)** refers to the following: GO:0000003 (reproduction), GO:0003723 (RNA binding), GO:0003824 (catalytic activity), GO:0005215 (transporter activity), GO:0005488 (binding), GO:0005576 (extracellular region), GO:0005618 (cell wall), GO:0005622 (intracellular), GO:0005623 (cell), GO:0005737 (cytoplasm), GO:0006519 (cellular amino acid and derivative metabolic process), GO:0006810 (transport), GO:0006950 (response to stress), GO:0007275 (multicellular organismal development), GO:0008289 (lipid binding), GO:0009536 (plastid), GO:0009579 (thylakoid), GO:0009607 (response to biotic stimulus), GO:0009719 (response to endogenous stimulus), GO:0016020 (membrane), GO:0016787 (hydrolase activity), GO:0019748 (secondary metabolic process), GO:0022414 (reproductive process), GO:0030312 (external encapsulating structure), GO:0032501 (multicellular organismal process), GO:0032502 (developmental process), GO:0043226 (organelle), GO:0043227 (membrane-bounded organelle), GO:0043229 (intracellular organelle), GO:0043231 (intracellular membrane-bounded organelle), GO:0044281 (small molecule metabolic process), GO:0044424 (intracellular part), GO:0044444 (cytoplasmic part), GO:0044464 (cell part), GO:0050896 (response to stimulus), GO:0051179 (localization), GO:0051234 (establishment of localization), GO:0065008 (regulation of biological quality).
