# Supplementary material for: Identification and molecular characterization of tissue-preferred rice genes and their upstream regularly sequences on a genome-wide level
Source: BMC Plant Biol. 2014 Nov 27;14:331. doi: 10.1186/s12870-014-0331-2 (PMC4248441; doi:10.1186/s12870-014-0331-2)
Supplement: Additional file 7: — GUS gene expression patterns of transgenic lines carrying a URS:: GUS cassette shown by qRT-PCR analysis. [file 12870_2014_331_MOESM7_ESM.pdf]

Additional data file 7

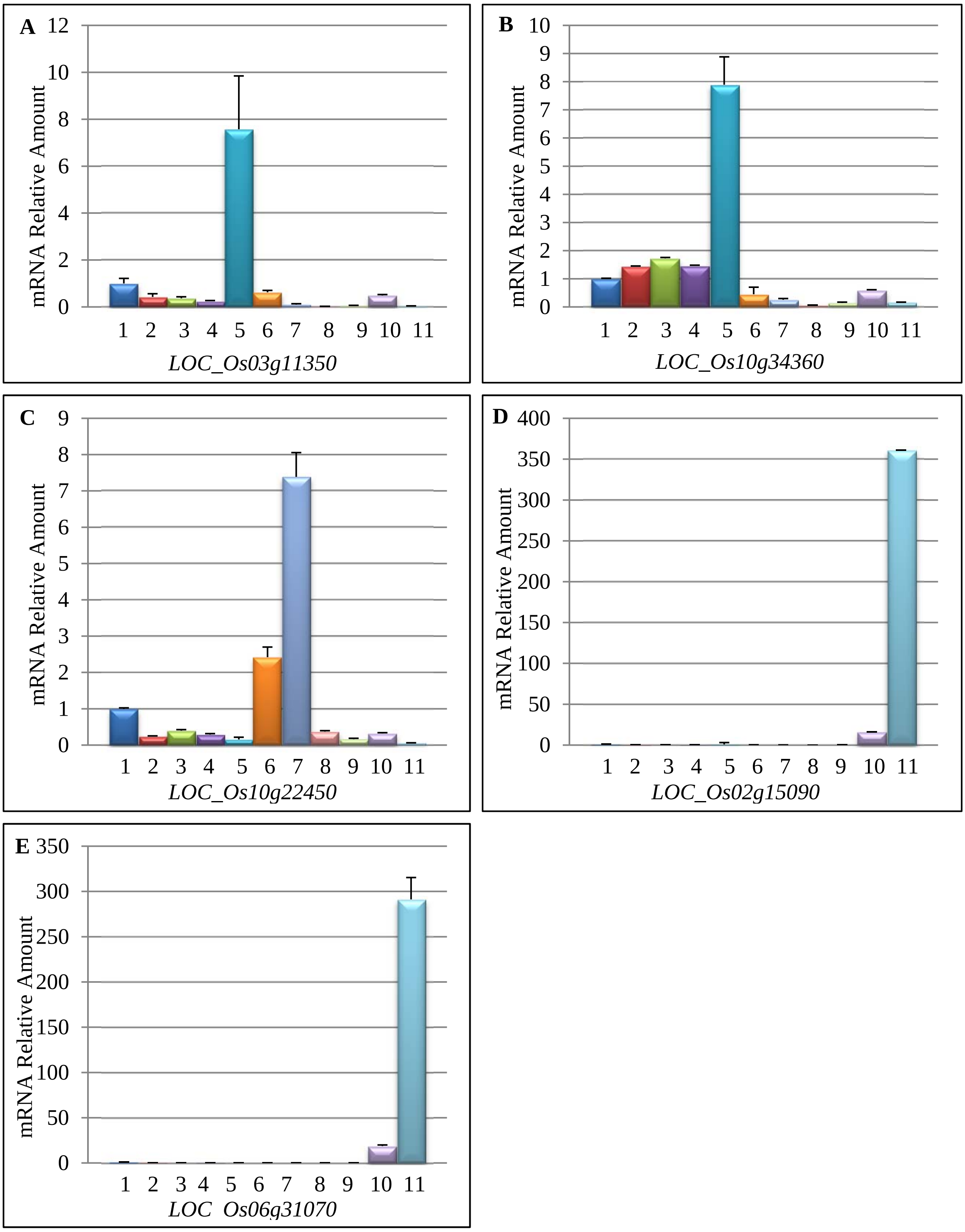

Additional data file 7. *GUS* gene expression patterns of transgenic lines carrying a URS::*GUS* cassette shown by qRT-PCR analysis.

The mRNA relative amount was calculated as described in the section ‘‘Methods’’. (A) to (E) showed the expression patterns of *GUS* genes in 5 URS::*GUS* transgenic lines. The total RNA samples prepared from a total of 11 tissues at different developmental stages were used for qRT-PCR. These tissues were listed as below: 1, two-week old leaves; 2, two-month old leaves; 3, two-week old roots; 4, two-month old roots; 5, 0-5cm long panicles; 6, 5-10cm long panicles; 7, more than 10cm long panicles; 8, opening panicles; 9, flowering panicles; 10, milky seeds; and 11, mature seeds.
