# Supplementary material for: Identification and molecular characterization of tissue-preferred rice genes and their upstream regularly sequences on a genome-wide level
Source: BMC Plant Biol. 2014 Nov 27;14:331. doi: 10.1186/s12870-014-0331-2 (PMC4248441; doi:10.1186/s12870-014-0331-2)
Supplement: Additional file 8: — Overrepresented URS motifs. [file 12870_2014_331_MOESM8_ESM.pdf]

Additional data file 8

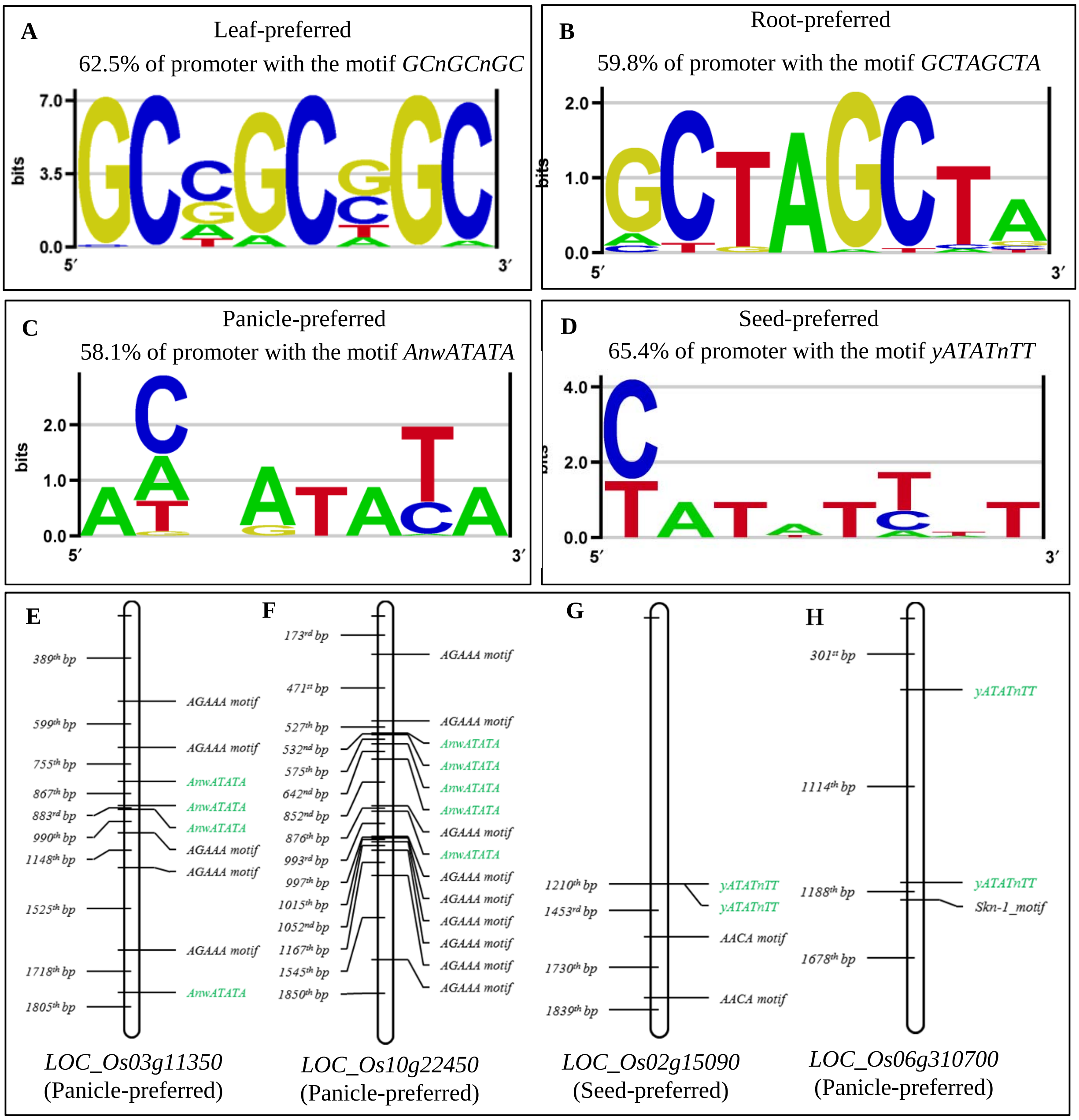

Additional data file 8. Overrepresented upstream regularly sequence motifs.

Upstream regularly sequences (URSs) were achieved from the upstream of start codon of each gene and were then submitted to the BioProspector program (Liu et al. 2001) to detect overrepresented motifs. The above logos were generated by submitting the detected overpresented motifs to the enoLOGOS program (Workman et al. 2005). (A) to (D) indicate the overrepresented URS motifs from leaf-preferred, root-preferred, panicle-preferred and seed-preferred genes, respectively. y=C/T; n=G/A/C/T; w=A/T. (E) to (G) Motif localization of two panicle-preferred and two seed-preferred URSs from four genes. Motif positions were indicated in left side and motif names were shown in right side. Green and black fonts in motif names indicated overrepresented and known motifs, respectively. Only panicle-related or seed-related known motifs were listed.
